# Supplementary material for: Selective Inhibition of Pulmonary Vein Excitability by Constitutively Active GIRK Channels Blockade in Rats
Source: Int J Mol Sci. 2023 Sep 4;24(17):13629. doi: 10.3390/ijms241713629 (PMC10487709; doi:10.3390/ijms241713629)
Supplement: Supplementary file 1 [file ijms-24-13629-s001.zip › ijms-2544748-supplementary.pdf]

Supplemental data

1. Table S1. Effects of 10^{-6}M carbachol (CCh) and 150 nM tertiapin-Q (TQ) upon spontaneous action potentials recorded from the sino-atrial node of the rat. Each recording was obtained first under basal condition, then during the superfusion of carbachol and finally following the addition of tertiapin-Q in the continued presence of carbachol. Results were obtained from 7 different preparations in which intracellular microelectrode recordings were maintained for each of the experimental conditions. P values in italics are given for statistical comparisons between data obtained under basal and 10^{-6}M CCh conditions and between 10-6M CCh conditions in the absence or presence of TQ.

	**Basal**		**10^{-6}M CCh**		**10^{-6}M CCh + TQ**
**Frequency (Hz)**	4.8 ± 0.2	*P<0.001*	2.6 ± 0.2	*P=0.004*	3.6 ± 0.2
**Phase 4 (ms)**	86 ± 5	*P<0.001*	243 ± 21	*P=0.012*	158 ± 19
**APD_{90} (ms)**	72.0 ± 2.5	*P<0.001*	54.1 ± 1.6	*P=0.001*	63.3 ± 1.5

2. Failure of anion current blockers to reverse α-adrenergic depolarization of the rat PV.

The results of Egorov et al. **[44,45]** showed that longitudinal stretch of the rat PV resulted in the depolarization of the membrane potential by the activation of the I_{swell} anion current. Here we describe experiments that tested whether the anion current blockers DIDS and DCPIB would reverse depolarization of the PV membrane potential evoked by the α1-adrenergic agonist cirazoline **[3,27]**.

Results

In these experiments, recordings were confined to the PV. The quiescent membrane potential (Figure 6 and **[27]**) was recorded in the absence of electrical stimulation with single intracellular microelectrodes as described in the materials and methods.

Figure S1 shows that from a membrane potential of -61.8±1.4 mV the superfusion of 1 µM cirazoline evoked a depolarization to -31.0 ± 1.3 mV (P<0.001; n=5). The addition of 200 µM DIDS in the continued presence of cirazoline had no significant effect, with a membrane potential of -31.0 ± 2.8 mV (P=0.98). The continued viability of these preparations was shown when DIDS was replaced by 1 µM carbachol in the continued presence of cirazoline and this evoked a hyperpolarization to -73.6±1.1 mV (P<0.001).

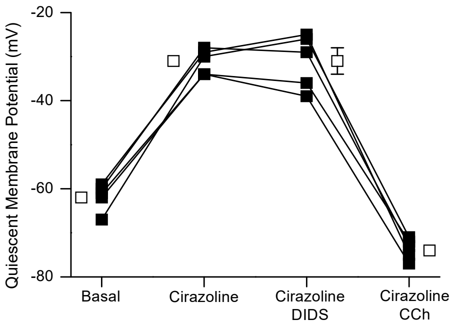

Figure S1: The effects of cirazoline (1 µM), DIDS (200 µM) and carbachol (CCh, 1 µM) upon the quiescent membrane potential of the PV. Data points linked by lines represent results obtained from individual microelectrode penetrations in the PV of five different preparations. Open symbols represent mean values. Vertical bars represent SEM values, shown when these were larger than the symbols.

Figure S2 illustrates a similar series of experiments where the I_{swell} blocker DCPIB was tested. In this series, AP were recorded in the PV following 5 Hz electrical stimulation of the LA wall in the same preparations. Under basal conditions the diastolic membrane potential was -71.5±0.9 mV (n=4). The superfusion of 1 µM cirazoline evoked a depolarization to -40.1±1.1 mV (P<0.001; n=5). In the continued presence of 1 µM cirazoline the addition of 10 µM DCPIB led to a slight but significant further depolarization to -35.2±1.0 mV (P=0.01; n=5). In this series, the continued viability of the preparations was tested by the replacement of DCPIB with 2 µM of the α-adrenergic antagonist prazosin in the continued presence of 1 µM cirazoline. This evoked a hyperpolarization to -67.5±1.2 mV (P<0.001; n=4).

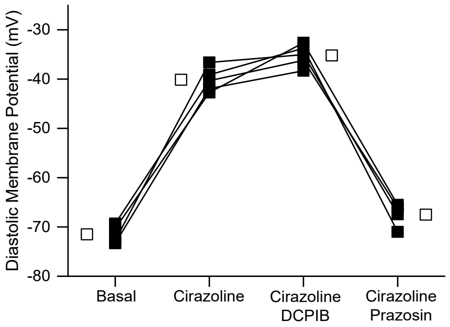

Figure S2: The effects of cirazoline (1 µM), DCPIB (10 µM) and prazosin (2 µM) upon the diastolic membrane potential recorded in the PV. Action potentials had been evoked by 5 Hz electrical stimulation applied to the LA in each preparation. Data points linked by lines represent results obtained from individual microelectrode penetrations. Open symbols represent mean values. In this set SEM values were all smaller than the symbols.

The fact that DCPIB led to a further depolarization in the continued presence of cirazoline led us to test the effects of DCPIB alone upon AP in the PV (Figure S3). In each of 4 different preparations 10 µM DCPIB provoked a depolarization from -71.7±2.2 mV to -55.9±1.8 mV (P=0.002). At the same time over-shooting AP were reduced from +14.6±2.1 mV (AP Peak) to small electrotonic waves at -49.0±3.5 mV (P<0.001). In the continued presence of 10 µM DCPIB, the addition of 1 µM carbachol resulted in a hyperpolarization to -73.1±2.4 mV (P=0.001) and the recovery of fully over-shooting AP to +17.0±2.6 mV (P<0.001).

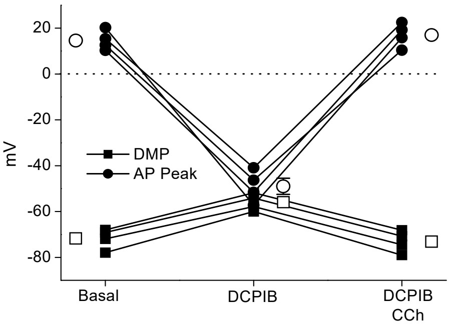

Figure S3: The effects of 10 µM DCPIB upon PV action potentials. Action potentials had been evoked by 5 Hz electrical stimulation applied to the LA in each preparation. Diastolic membrane potential (DMP, filled squares) and action potential peak voltage (AP Peak, filled circles) were first recorded under basal conditions, then in the presence of 10 µM DCPIB and finally following the addition of 10 µM carbachol (CCh) in the continued presence of DCPIB. Data points linked by lines represent the results obtained from individual microelectrode penetrations in the PV of four separate preparations. Open symbols represent mean values. SEM bars are shown when these were larger than the symbols.

Conclusions.

Neither DIDS (Supplementary Figure S1) nor DCPIB (Supplementary Figure S2) reversed the depolarization of the PV membrane potential evoked by cirazoline. In fact DCPIB significantly further depolarized the PV

membrane potential. Neither of these results support the hypothesis that α1-adrenergic receptor activation was provoking depolarization of the PV membrane potential by the activation of an anionic membrane current.

DCPIB alone provoked depolarization of the PV membrane potential and the reduction of electrically evoked action potentials to small electronic waves (Supplementary Figure S3). This was reversed by carbachol. These results are similar to those that we show to be evoked in the PV by the GIRK channel blocker TQ (Figures 3 and 4). Although DCPIB is a clear blocker of I_{swell} **[44,45]**, Deng et al. **[42]** found it to also be an inhibitor of a variety of inwardly rectified K^+ ion channels, including GIRK.
